# Supplementary material for: Identifying and assessing nonlinear drivers of capelin and Atlantic cod population dynamics using empirical dynamic modelling (EDM) scenario exploration
Source: PLoS One. 2025 Dec 19;20(12):e0339407. doi: 10.1371/journal.pone.0339407 (PMC12716747; doi:10.1371/journal.pone.0339407)
Supplement: S1 File — (DOCX) [file pone.0339407.s001.docx]

**Supporting Information: Raw data and individual scenario exploration figures**

**
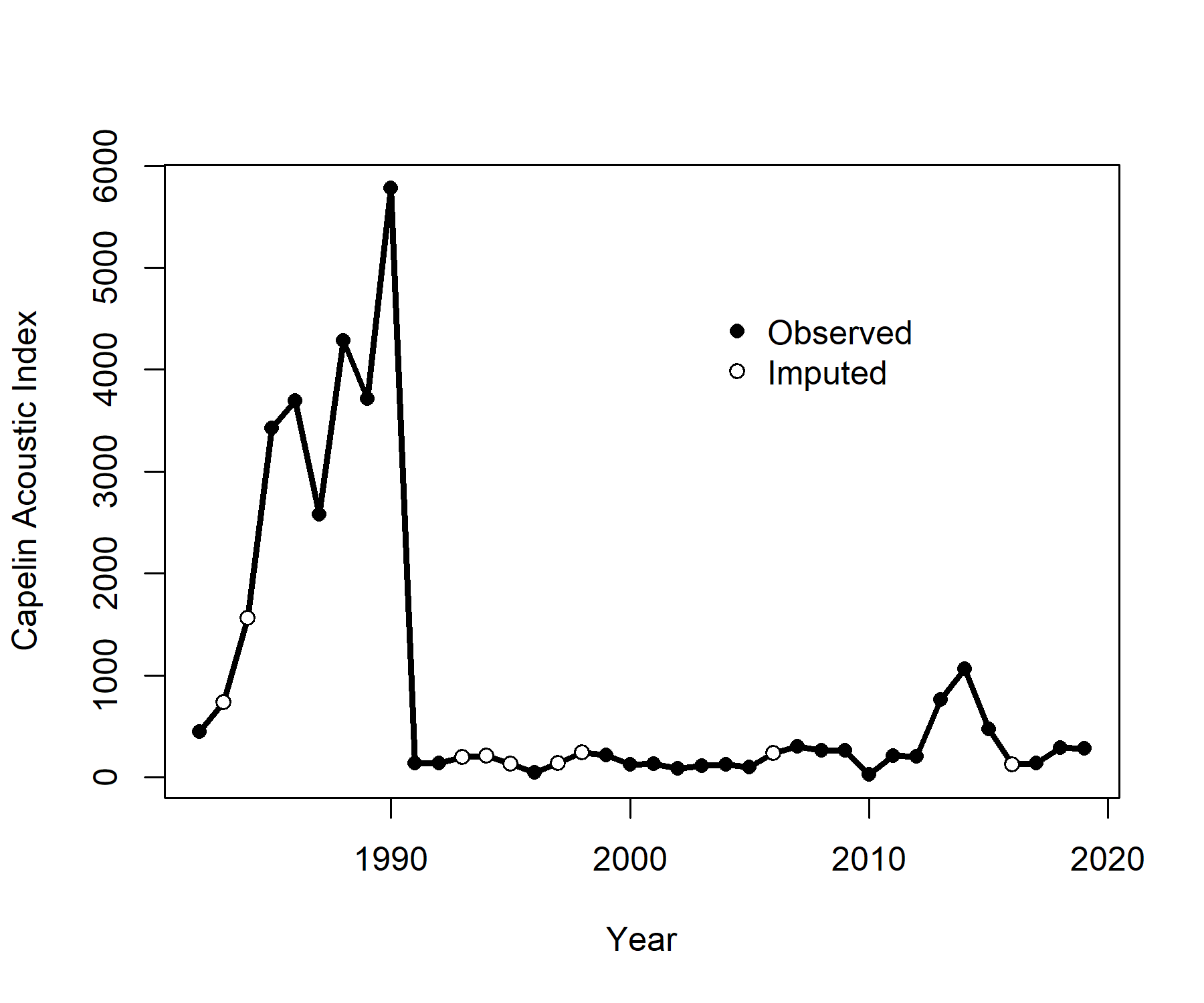
**

**Figure S1** Time series of the raw capelin acoustic index, with imputed values identified by open points. Imputed values after 1990 were calculated by interpolation of a Gaussian process regression on the time series from 1991 to 2019. Imputed values before 1990 were calculated by interpolation of a Gaussian process regression using the full time series.


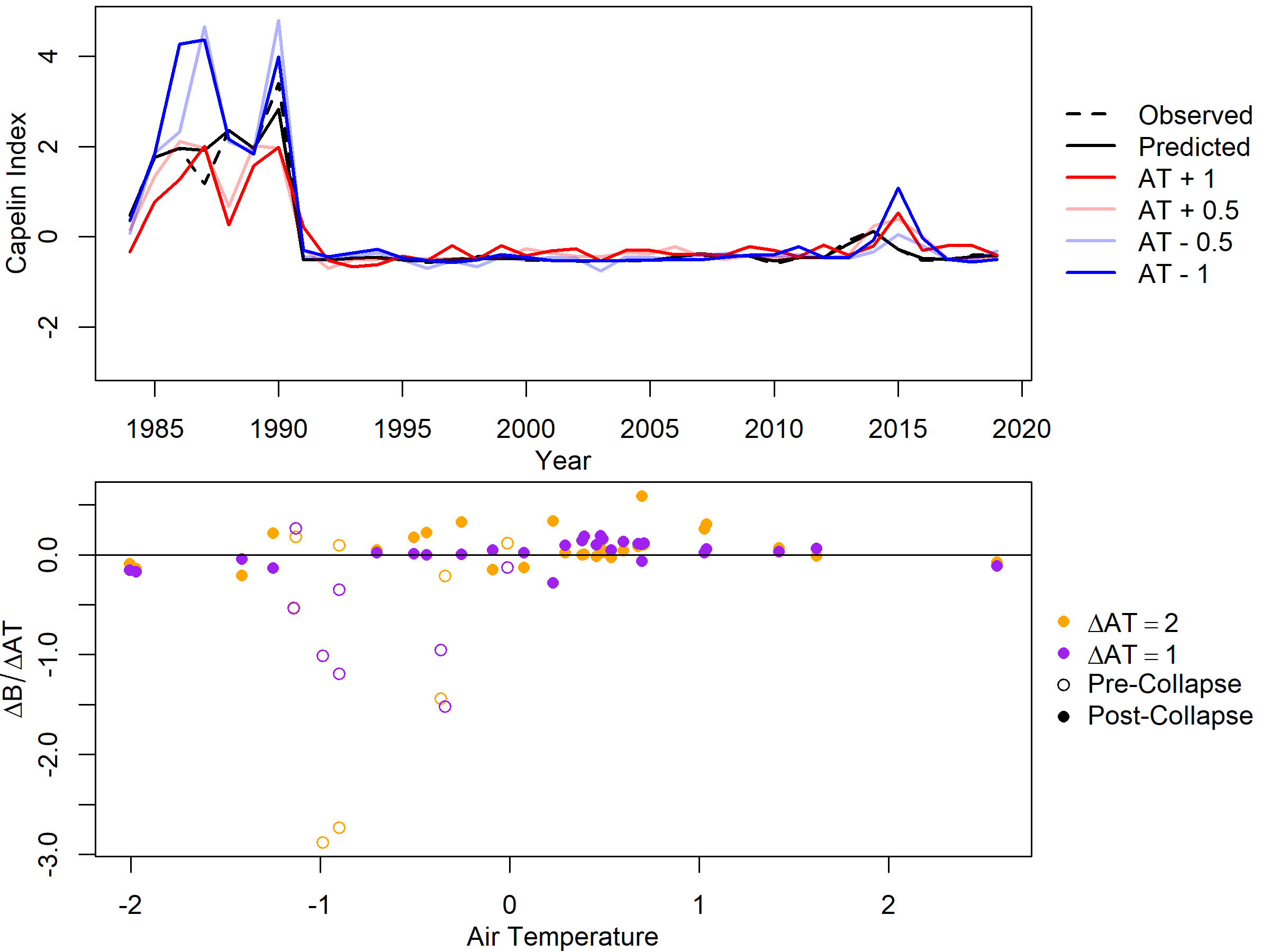


**Figure S2** Time series of the observed capelin acoustic index, S-Map predicted capelin acoustic index with air temperature, and predicted changes in the capelin acoustic index using S-Map scenario exploration with air temperature perturbed positively and negatively by a half standard deviation and a full standard deviation from 1984-2019 (top), and scatterplot of the difference between positive perturbation predictions and negative perturbation predictions for each year in the time series plotted against normalized air temperature.

**
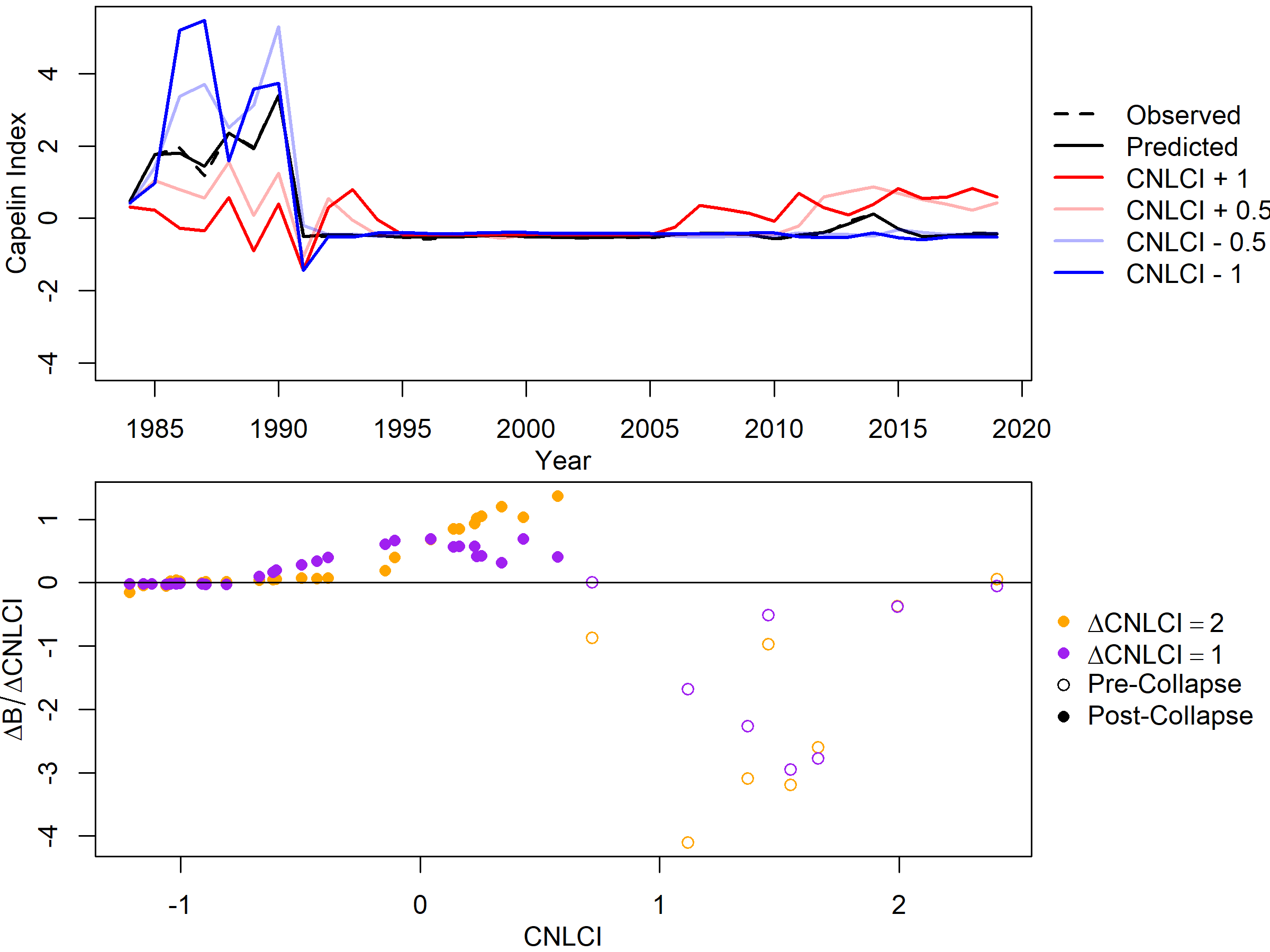
**

**Figure S3** Time series of the observed capelin acoustic index, S-Map predicted capelin acoustic index with CNLCI, and predicted changes in the capelin acoustic index using S-Map scenario exploration with CNLCI perturbed positively and negatively by a half standard deviation and a full standard deviation from 1984-2019 (top), and scatterplot of the difference between positive perturbation predictions and negative perturbation predictions for each year in the time series plotted against normalized CNLCI.

**
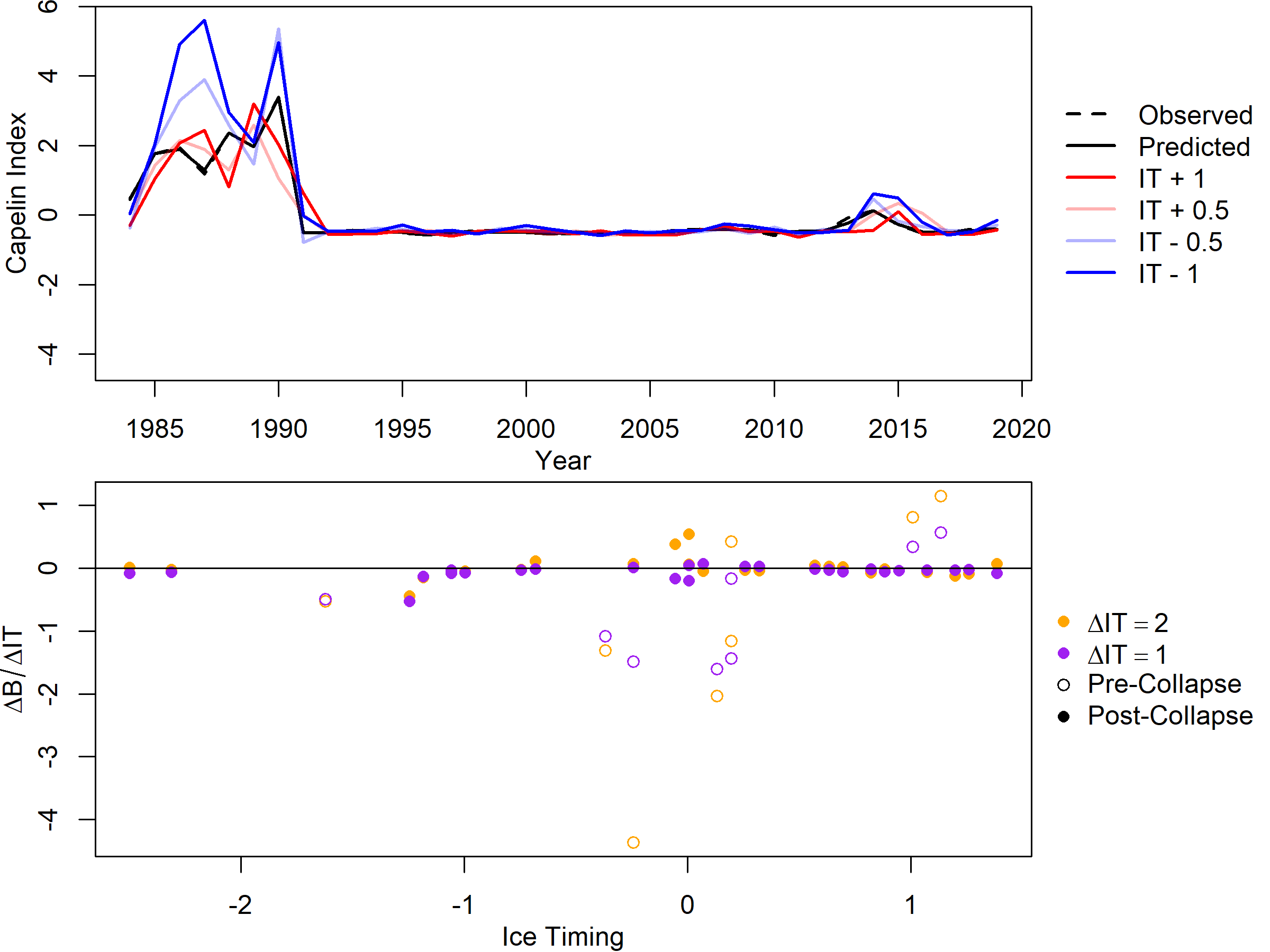
**

**Figure S4** Time series of the observed capelin acoustic index, S-Map predicted capelin acoustic index with ice timing, and predicted changes in the capelin acoustic index using S-Map scenario exploration with ice timing perturbed positively and negatively by a half standard deviation and a full standard deviation from 1984-2019 (top), and scatterplot of the difference between positive perturbation predictions and negative perturbation predictions for each year in the time series plotted against normalized ice timing.

**
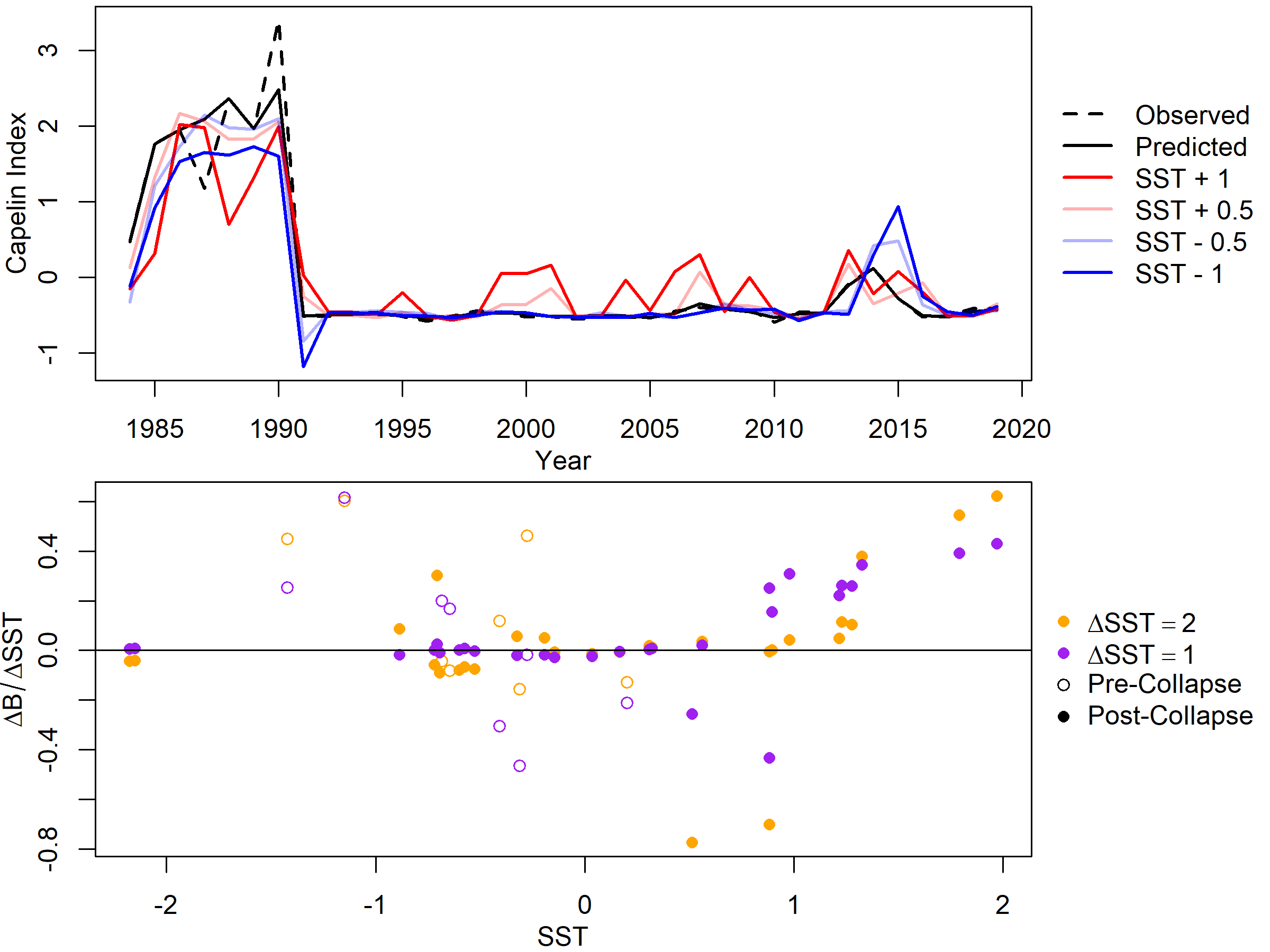
**

**Figure S5** Time series of the observed capelin acoustic index, S-Map predicted capelin acoustic index with SST, and predicted changes in the capelin acoustic index using S-Map scenario exploration with SST perturbed positively and negatively by a half standard deviation and a full standard deviation from 1984-2019 (top), and scatterplot of the difference between positive perturbation predictions and negative perturbation predictions for each year in the time series plotted against normalized SST.

**
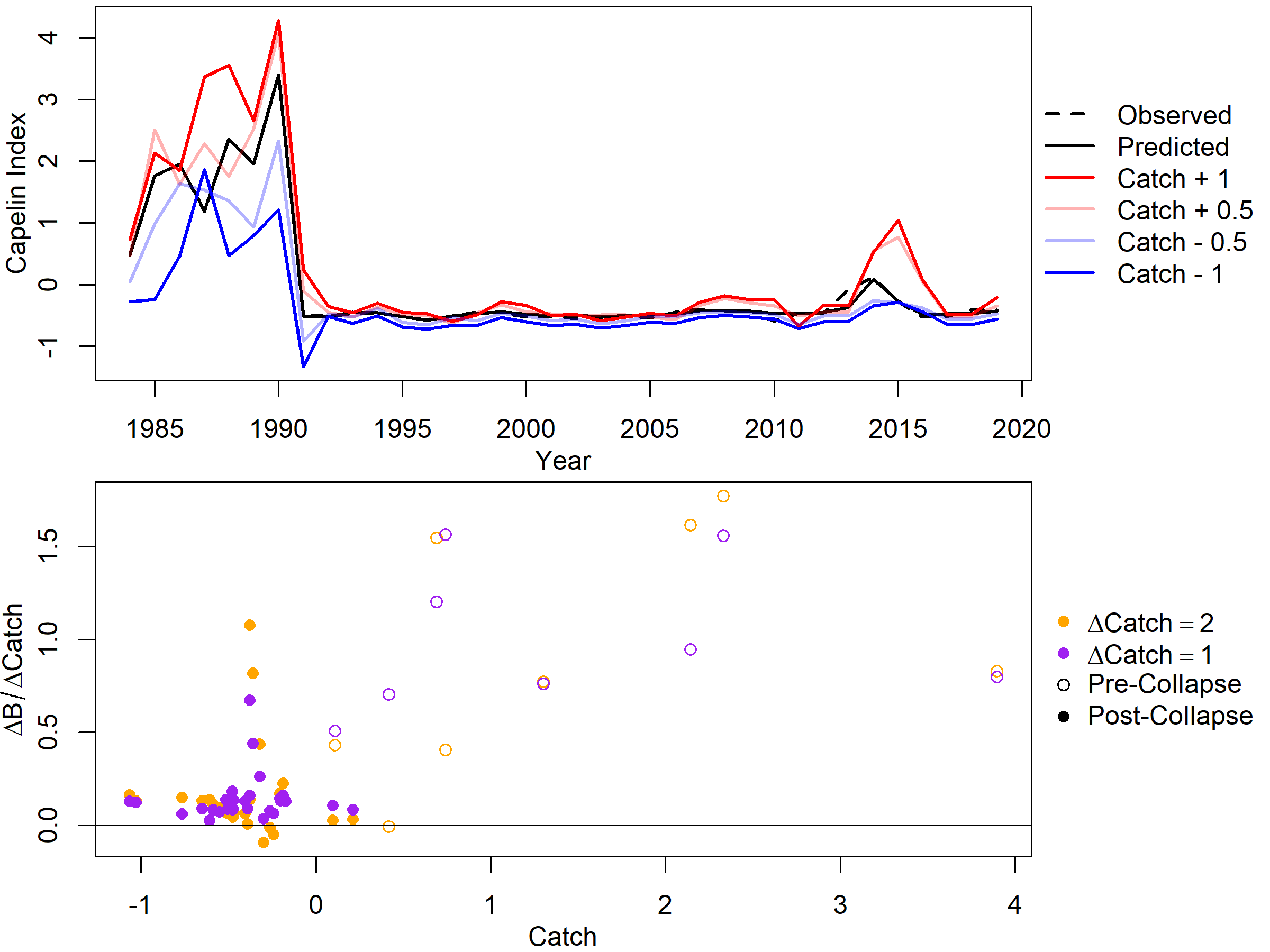
**

**Figure S6** Time series of the observed capelin acoustic index, S-Map predicted capelin acoustic index with capelin catch, and predicted changes in the capelin acoustic index using S-Map scenario exploration with capelin catch perturbed positively and negatively by a half standard deviation and a full standard deviation from 1984-2019 (top), and scatterplot of the difference between positive perturbation predictions and negative perturbation predictions for each year in the time series plotted against normalized capelin catch.

**
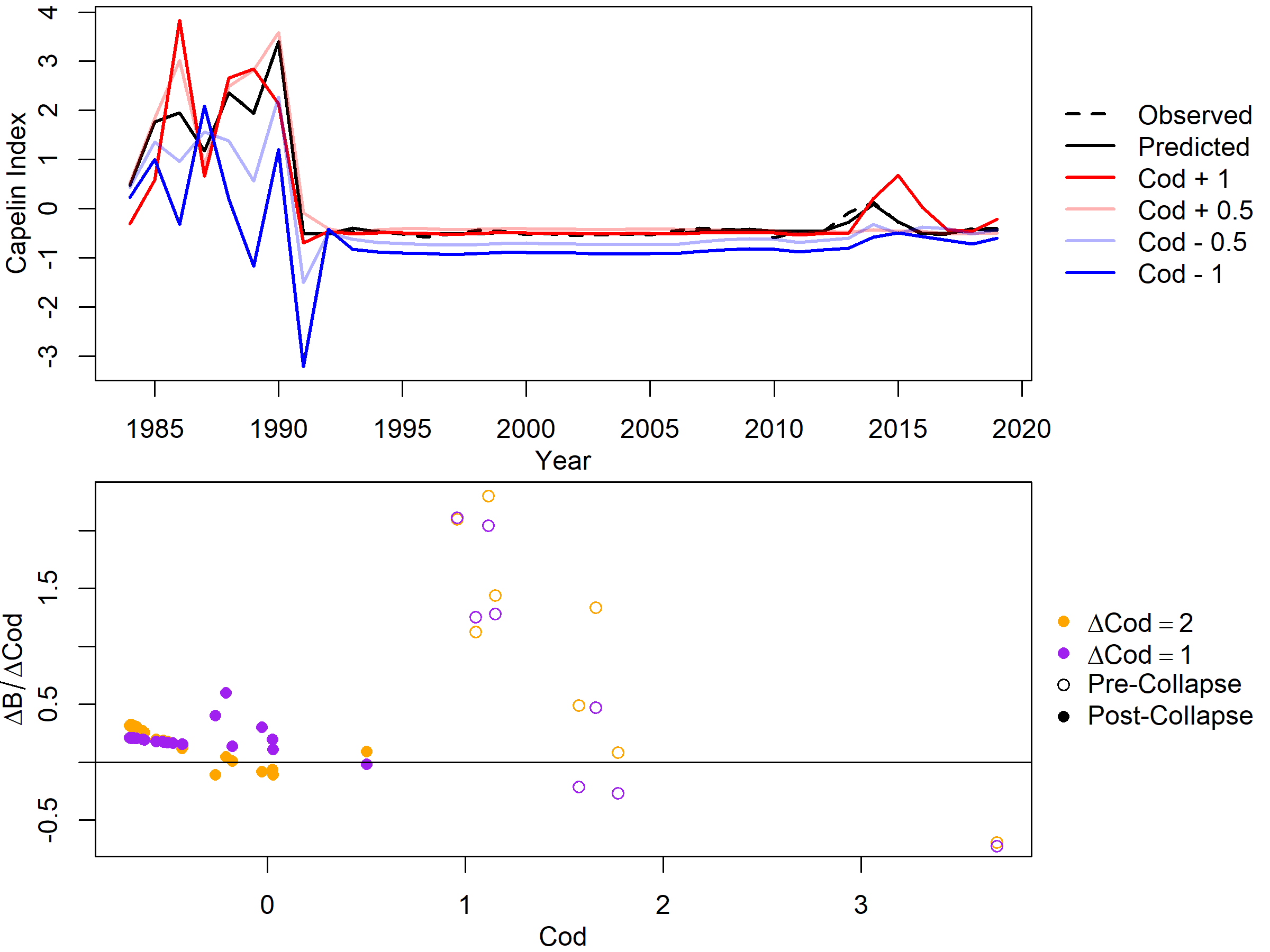
**

**Figure S7** Time series of the observed capelin acoustic index, S-Map predicted capelin acoustic index with the Atlantic cod bottom trawl index, and predicted changes in the capelin acoustic index using S-Map scenario exploration with cod perturbed positively and negatively by a half standard deviation and a full standard deviation from 1984-2019 (top), and scatterplot of the difference between positive perturbation predictions and negative perturbation predictions for each year in the time series plotted against the normalized Atlantic cod bottom trawl index.

**
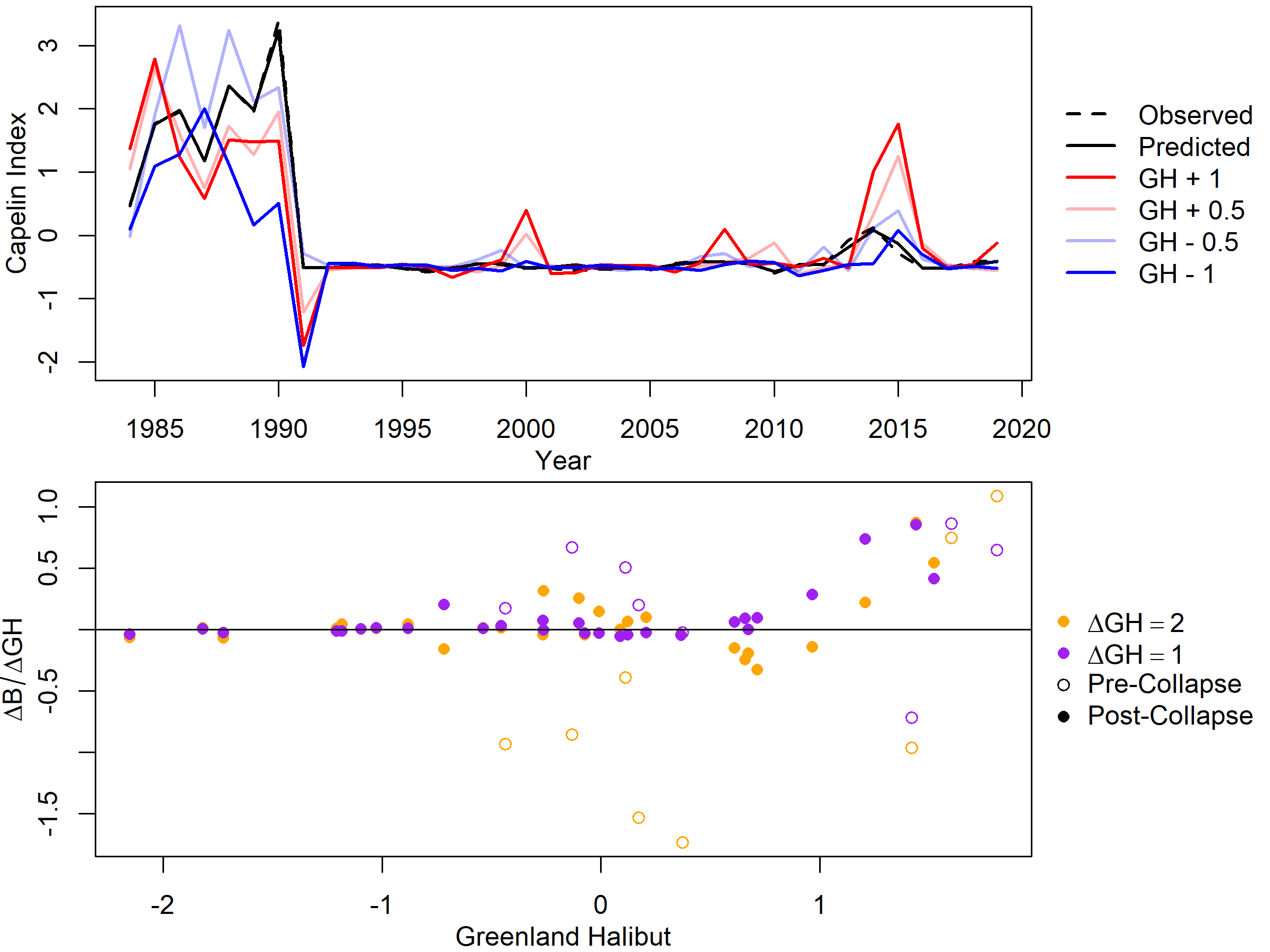
**

**Figure S8** Time series of the observed capelin acoustic index, S-Map predicted capelin acoustic index with the Greenland halibut bottom trawl index, and predicted changes in the capelin acoustic index using S-Map scenario exploration with Greenland halibut perturbed positively and negatively by a half standard deviation and a full standard deviation from 1984-2019 (top), and scatterplot of the difference between positive perturbation predictions and negative perturbation predictions for each year in the time series plotted against the normalized Greenland halibut bottom trawl index.

**
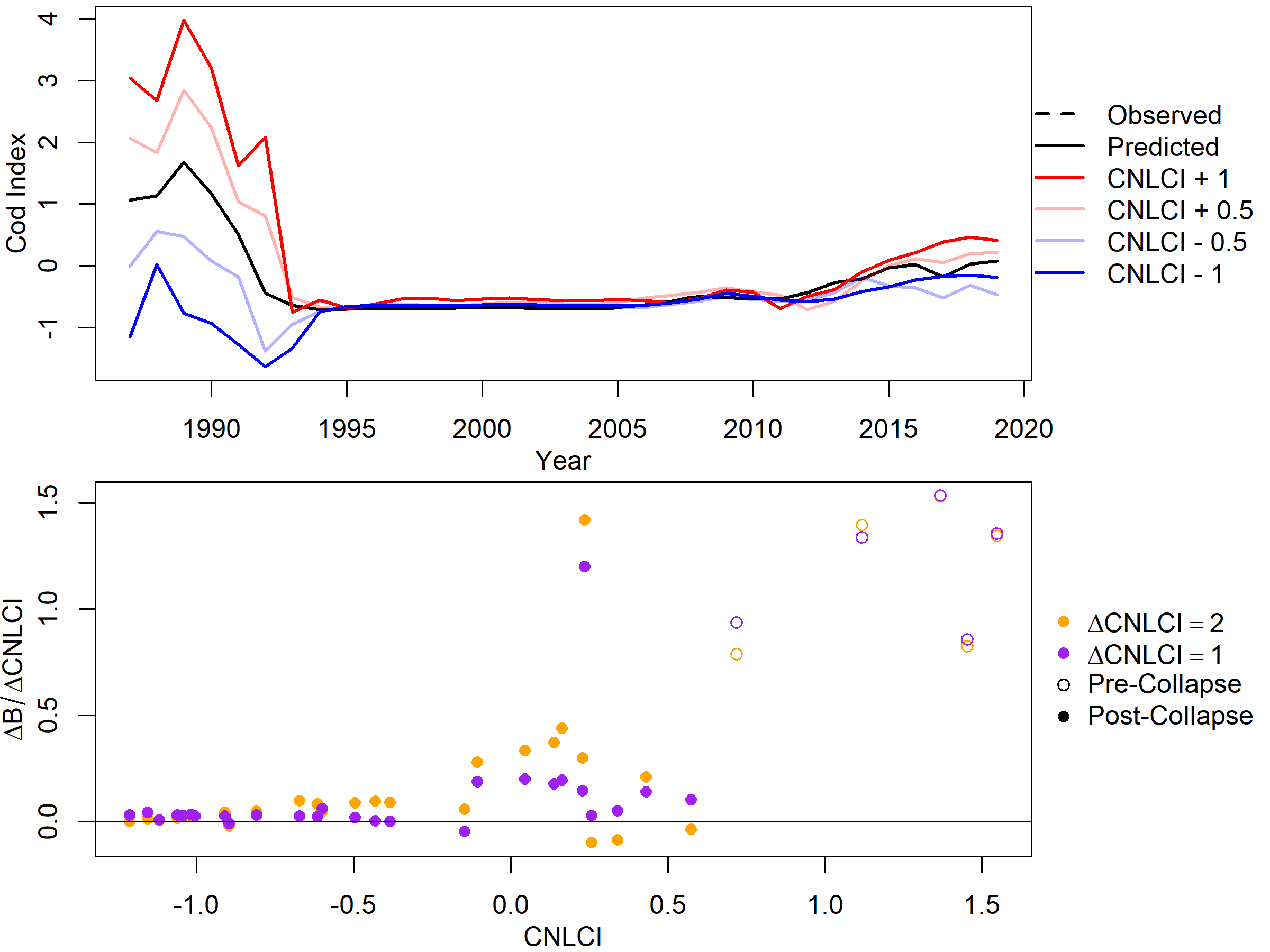
**

**Figure S9** Time series of the observed Atlantic cod bottom trawl index, S-Map predicted Atlantic cod bottom trawl index with CNLCI, and predicted changes in the Atlantic cod bottom trawl index using S-Map scenario exploration with CNLCI perturbed positively and negatively by a half standard deviation and a full standard deviation from 1984-2019 (top), and scatterplot of the difference between positive perturbation predictions and negative perturbation predictions for each year in the time series plotted against normalized CNLCI.

**
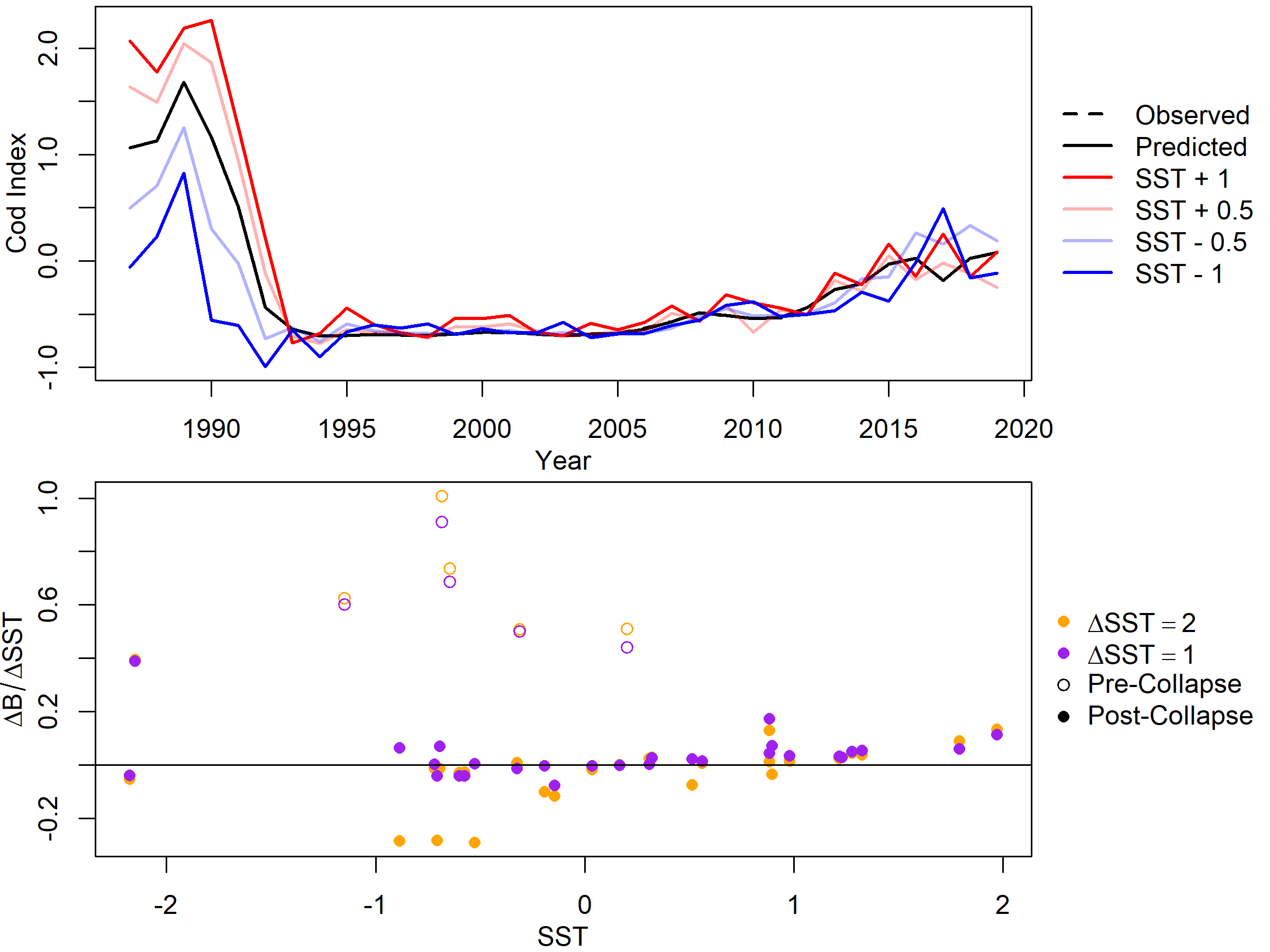
**

**Figure S10** Time series of the observed Atlantic cod bottom trawl index, S-Map predicted Atlantic cod bottom trawl index with SST, and predicted changes in the Atlantic cod bottom trawl index using S-Map scenario exploration with SST perturbed positively and negatively by a half standard deviation and a full standard deviation from 1984-2019 (top), and scatterplot of the difference between positive perturbation predictions and negative perturbation predictions for each year in the time series plotted against normalized SST.

**
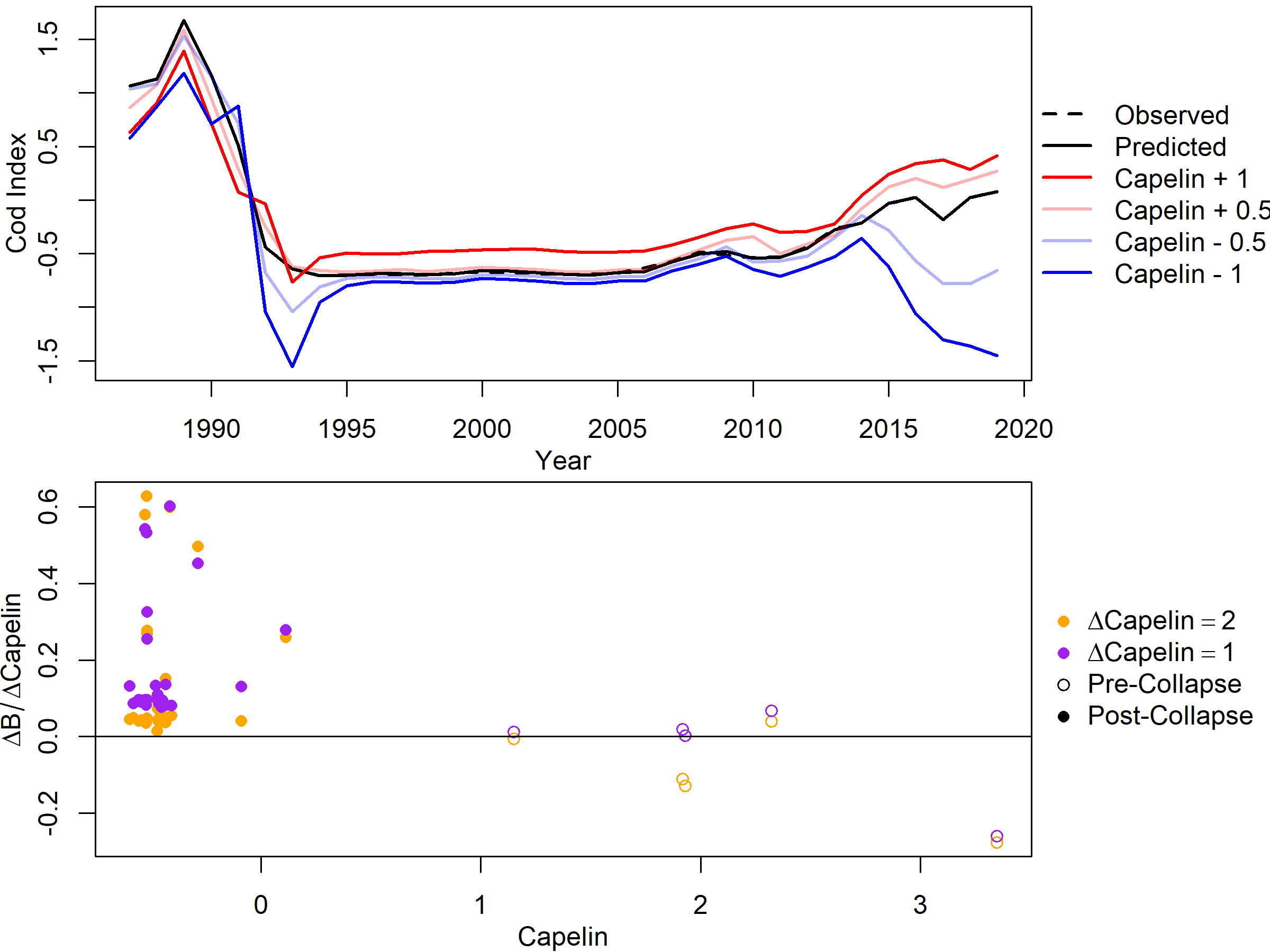
**

**Figure S11** Time series of the observed Atlantic cod bottom trawl index, S-Map predicted Atlantic cod bottom trawl index with the capelin acoustic index, and predicted changes in the Atlantic cod bottom trawl index using S-Map scenario exploration with capelin perturbed positively and negatively by a half standard deviation and a full standard deviation from 1984-2019 (top), and scatterplot of the difference between positive perturbation predictions and negative perturbation predictions for each year in the time series plotted against the normalized capelin acoustic index.

**
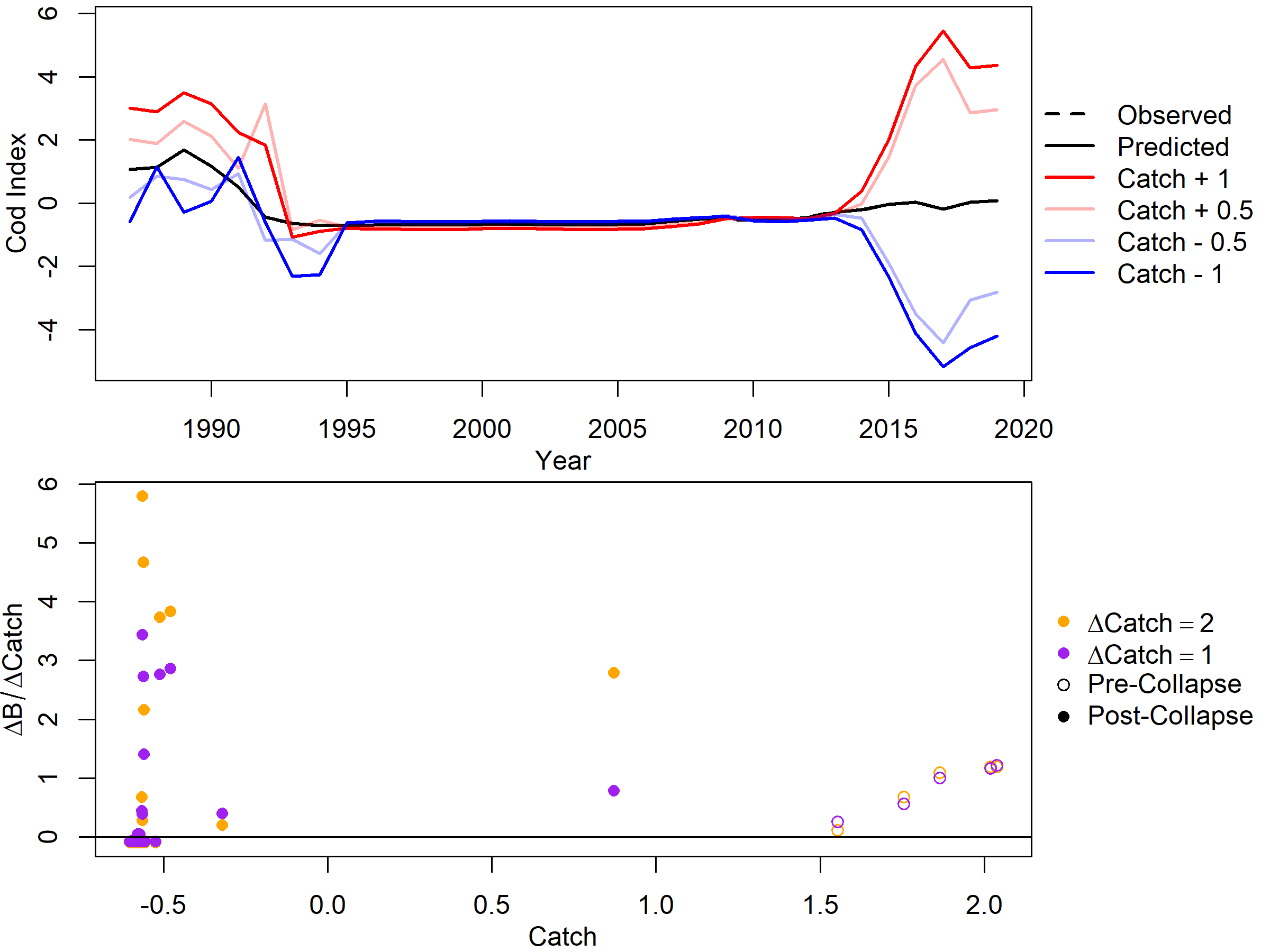
**

**Figure S12** Time series of the observed Atlantic cod bottom trawl index, S-Map predicted Atlantic cod bottom trawl index with cod catch, and predicted changes in the Atlantic cod bottom trawl index using S-Map scenario exploration with cod catch perturbed positively and negatively by a half standard deviation and a full standard deviation from 1984-2019 (top), and scatterplot of the difference between positive perturbation predictions and negative perturbation predictions for each year in the time series plotted against normalized cod catch.

**
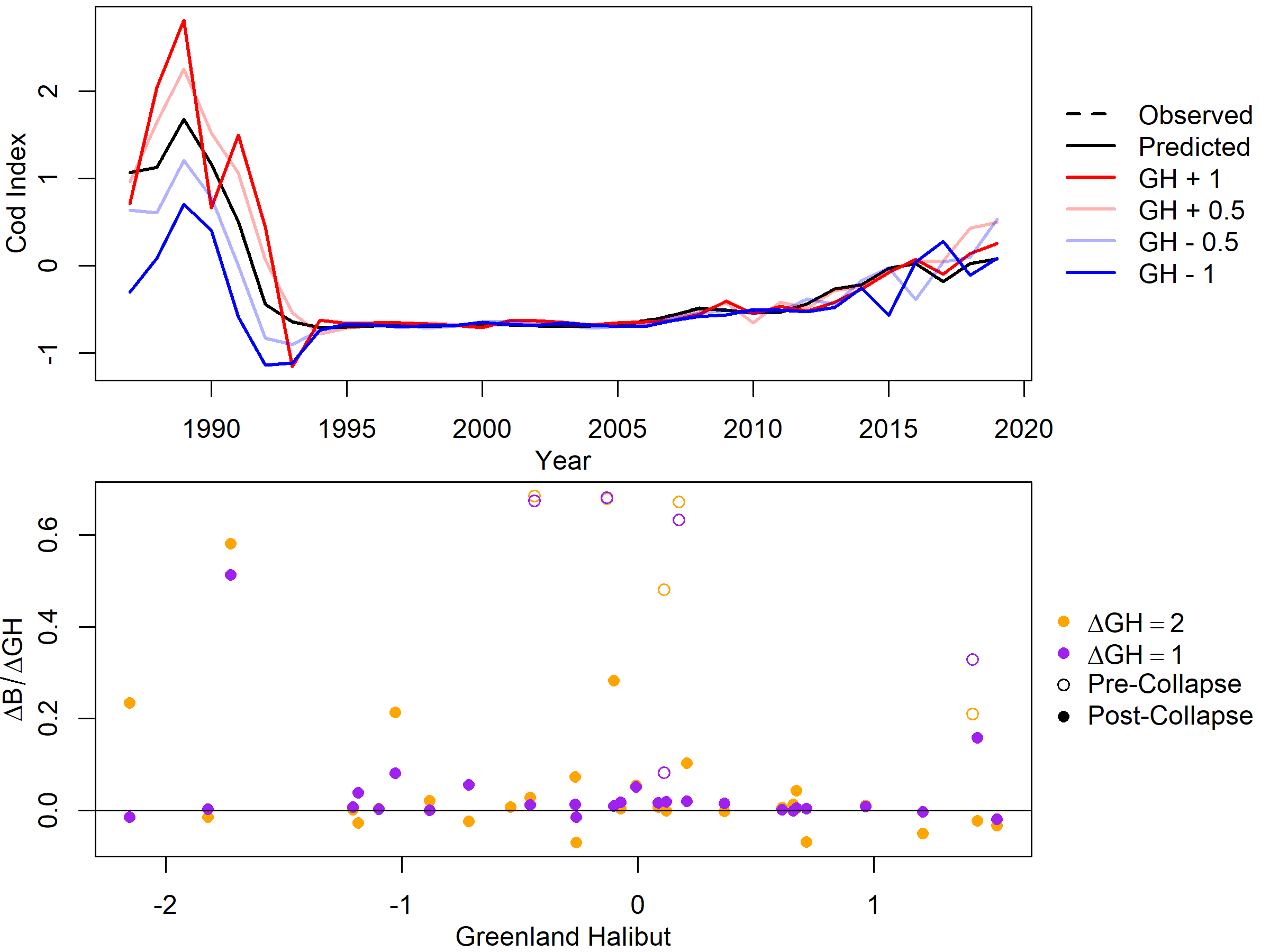
**

**Figure S13** Time series of the observed Atlantic cod bottom trawl index, S-Map predicted Atlantic cod bottom trawl index with the Greenland halibut bottom trawl index, and predicted changes in the Atlantic cod bottom trawl index using S-Map scenario exploration with Greenland halibut perturbed positively and negatively by a half standard deviation and a full standard deviation from 1984-2019 (top), and scatterplot of the difference between positive perturbation predictions and negative perturbation predictions for each year in the time series plotted against the normalized Greenland halibut bottom trawl index.
