# Supplementary material for: Identifying and assessing nonlinear drivers of capelin and Atlantic cod population dynamics using empirical dynamic modelling (EDM) scenario exploration
Source: PLoS One. 2025 Dec 19;20(12):e0339407. doi: 10.1371/journal.pone.0339407 (PMC12716747; doi:10.1371/journal.pone.0339407)
Supplement: Attachment — Submitted filename: EDM_Comments2Authors.pdf [file pone.0339407.s002.pdf]

Review Report of Manuscript "Empirical Dynamic Modelling of Capelin and Cod Population Dynamics on the Newfoundland Shelf"

Overview

The manuscript investigates the nonlinear drivers of capelin (*Mallotus villosus*) and Atlantic cod (*Gadus morhua*) population dynamics on the Newfoundland Shelf, employing Empirical Dynamic Modelling (EDM) and scenario exploration. The study aims to identify ecological and climatic drivers of biomass fluctuations and evaluate nonlinear responses to perturbations in these drivers. The paper is generally well written, with a clear structure and a balanced discussion of the results.

I consider the general focus on nonlinear, state-dependent dynamics as timely and highly relevant, given ongoing interest in data-driven fisheries assessment under climate change. Furthermore, the application of EDM scenario exploration to capelin and cod is innovative and provides insights beyond traditional linear modelling approaches.

Having said that, I find several methodological and interpretive issues, which limit the strength of the manuscript and the conclusions. I am convinced that addressing these concerns would substantially improve the manuscript's impact. I briefly discuss these concerns under two broad (Major/Minor) headings.

Major Comments

1. **Causality vs. synchrony.** The most critical issue concerns interpretation of causal drivers. I am not convinced, for example, that the positive relationships between catch and biomass, or cod biomass driving capelin biomass, are more than mere statistical artifacts of synchrony. At least, no strong analysis is provided to proof that such relationships reflect true ecological processes. While the manuscript acknowledges this limitation, the conclusions at times still overstate causality. Stronger emphasis on this distinction is needed throughout.

2. **Treatment of catch as a driver.** Including catch as a covariate is problematic, since it is inherently linked to biomass. This risks circular reasoning and confounds inference. More appropriate proxies such as fishing effort, standardized CPUE, or estimates of fishing mortality could be viable alternatives to be considered.

3. **Single-covariate perturbations.** The scenario exploration presented involves perturbation of one covariate at a time. However, this approach ignores the joint effects of multiple interacting drivers. Ecosystem dynamics are inherently multivariate, and this simplification may obscure important synergistic or antagonistic interactions. Exploring multivariate perturbations would strengthen the analysis.

4. **Temporal coverage and data limitations.** The dataset does not cover earlier periods of high fishing pressure (1960s–1970s). I fear that this could limit the ability to detect true catch–biomass relationships. Extending the dataset to include earlier decades if possible, will help better capture periods of high exploitation, which could be contrasted with post-moratorium dynamics. The authors should discuss how their choice of data (exclusion) affects interpretation and whether supplementary data could be incorporated.

5. **Validation of model predictions.** The manuscript would benefit from a more rigor-

ous validation framework. For example, cross-validation, hindcasting, or comparisons with independent ecological knowledge could demonstrate whether scenario exploration produces reliable forecasts.

6. **Mechanistic interpretation.** While the discussion speculates on mechanisms such as thermal performance curves and match–mismatch dynamics, these remain untested. Without independent evidence, such mechanisms should be framed more cautiously, or ideally tested through complementary modeling approaches.

Minor Comments

- **Terminology.** Ensure consistent use of “drivers,” “covariates,” and “indices,” as these terms are occasionally interchanged.
- **Figures.** Several figures are complex and could benefit from clearer labelling (e.g., distinguishing pre- and post-collapse periods more explicitly).
- **Table 1.** Consider highlighting significant CCM results more visually (e.g., bold or shading), as the asterisks are easy to miss.
- **Citations.** Some references to previous studies (e.g., cod–capelin bottom-up control) could be expanded to situate results more firmly in the existing literature.
- **Language.** The manuscript is generally clear, but occasional phrasing (e.g., “likely statistical artifacts”) could be expressed with more academic precision.
