# Supplementary material for: Identifying and assessing nonlinear drivers of capelin and Atlantic cod population dynamics using empirical dynamic modelling (EDM) scenario exploration
Source: PLoS One. 2025 Dec 19;20(12):e0339407. doi: 10.1371/journal.pone.0339407 (PMC12716747; doi:10.1371/journal.pone.0339407)
Supplement: Attachment — Submitted filename: Ch2_R2R1_F.docx [file pone.0339407.s003.docx]

Dear PLoS ONE Editorial Staff,

We appreciate your interest in our study, and your consideration for its publication in PLoS ONE. We found the reviewer’s comments to be thoughtful and beneficial in improving our revised manuscript. We have addressed each comment specifically below, with direct references to the revised manuscript where relevant. We are happy to continue working to improve the manuscript as necessary, and we look forward to hearing from you again in the near future.

Sincerely,

R.W. Steele, J. Gao, M. Koen-Alonso, and P.M. Regular

1. *Causality vs. synchrony. The most critical issue concerns interpretation of causal drivers. I am not convinced, for example, that the positive relationships between catch and biomass, or cod biomass driving capelin biomass, are more than mere statistical artifacts of synchrony. At least, no strong analysis is provided as proof that such relationships reflect true ecological processes. While the manuscript acknowledges this limitation, the conclusions at times still overstate causality. Stronger emphasis on this distinction is needed throughout.*

We agree with the reviewer and have strengthened our language surrounding synchrony and spurious drivers at lines 163-170, and throughout the revised manuscript.

2. *Treatment of catch as a driver. Including catch as a covariate is problematic, since it is inherently linked to biomass. This risks circular reasoning and confounds inference. More appropriate proxies such as fishing effort, standardized CPUE, or estimates of fishing mortality could be viable alternatives to be considered.*

We agree with the reviewer’s point that catch is a problematic covariate due to its inherent link to biomass, and resulting synchrony. We primarily include it in the paper to serve as a cautionary tale exemplifying a weakness of EDM scenario exploration when using covariates which are strongly driven by the target time series.

Of the reviewer’s suggestions, standardized CPUE also serves as a biomass index, and is likely to suffer the same issue. In an attempt to satisfy this concern, we attempted two new analyses – one using nominal harvest rate (i.e. catch/biomass), and one using total days fished across all gear types as reported in the NAFO STATLANT 21b database to serve as a crude metric of error.

The analysis of nominal harvest rate suffered the same issues as catch, and so we felt it would offer no additional value to the study and we did not include it. The analysis of effort did identify the expected top-down relationship for capelin, but largely appeared to reflect biomass for cod, similar to catch. Additionally, we are concerned with the quality of this effort data due to inconsistent reporting. Due to these concerns, we did not add the analysis of effort data to the revised manuscript. However, if the reviewers deem it valuable despite these concerns, we are not opposed to adding it in the future. We have attached preliminary fishing effort scenario exploration figures for capelin and cod below.

We further discuss issues with the use of catch as a driver at lines 167-169 and 217-222 of the revised manuscript.

**Figure R1** Preliminary scenario exploration of fishing effort (total days fished across all gear types) on capelin biomass, showing scenario exploration time series predictions (top) and ΔB/ΔX values (bottom) for +1SD (dark/filled red), +0.5SD (light/open red), -0.5SD (light/open blue), and -1SD (dark/open blue) perturbations of fishing effort.

**Figure R2** Preliminary scenario exploration of fishing effort (total days fished across all gear types) on cod biomass, showing scenario exploration time series predictions (top) and ΔB/ΔX values (bottom) for +1SD (dark/filled red), +0.5SD (light/open red), -0.5SD (light/open blue), and -1SD (dark/open blue) perturbations of fishing effort.

3. *Single-covariate perturbations. The scenario exploration presented involves perturbation of one covariate at a time. However, this approach ignores the joint effects of multiple interacting drivers. Ecosystem dynamics are inherently multivariate, and this simplification may obscure important synergistic or antagonistic interactions. Exploring multivariate perturbations would strengthen the analysis.*

We agree with the reviewer’s point that ecosystem dynamics are complex, and synergistic or antagonistic interactions between driving forces are likely to occur. However, realistically applying multivariate perturbations is challenging, as (1) the number of possible driver combinations to test grows rapidly with the number of drivers, and (2) the values of drivers are also inherently linked, meaning simple, uninformed perturbations such as those we use in this study applied to multiple covariates simultaneously are likely to result in unrealistic scenarios. We discuss this limitation in the revised manuscript at lines 233-247.

To bridge the gap between these two analyses, we have added an additional analysis to the paper where we perform multivariate scenario exploration on cod biomass using scenario exploration biomass predictions of capelin biomass as a second covariate alongside common climatic drivers of both species (CNLCI and SST). This ensures the perturbations of the climatic variables and capelin biomass can reasonably coexist. The results of this analysis can be found in Figure 4, and are discussed throughout the manuscript at lines 148-157, 204-215, and 337-351.

4. *Temporal coverage and data limitations. The dataset does not cover earlier periods of high fishing pressure (1960s–1970s). I fear that this could limit the ability to detect true catch–biomass relationships. Extending the dataset to include earlier decades if possible, will help better capture periods of high exploitation, which could be contrasted with post-moratorium dynamics. The authors should discuss how their choice of data (exclusion) affects interpretation and whether supplementary data could be incorporated.*

We agree with the reviewer in that it would be beneficial to be able to analyze earlier periods. However, in the capelin acoustic index and the fall bottom trawl survey, we have selected what we believe to be the most accurate biomass indices available for 2K3KL capelin and cod respectively, and we use the entirety of both datasets. As such, we are unable to extend the analysis into earlier periods. This could potentially be done by using less accurate biomass indices, but we prefer the quality of the time series used in our study. We discuss this decision and its consequences at lines 225-232 of the revised manuscript.

Regarding time series length, we also received this comment in the revision email:

*Rev 1 sees a danger of model overparameterization due to relatively short time series as compared to the number of pressures. While I for sure do not ask for longer time series, there is a need for you to explain why this is not a serious issue.*

Although we test many covariates in this study, we do not use multiple at once, with the exception of the new multivariate scenario exploration described in point 3 above. The greatest embedding dimension (i.e. number of model parameters) used in this study is 4, which we do not believe would be overparameterized given the length of our time series.

5. *Validation of model predictions. The manuscript would benefit from a more rigorous validation framework. For example, cross-validation, hindcasting, or comparisons with independent ecological knowledge could demonstrate whether scenario exploration produces reliable forecasts.*

Though we cannot directly validate scenario exploration results as they involve false perturbations, we agree with the reviewer that the manuscript would benefit from further validation. In response, we have calculated leave-one-out cross-validation prediction skill values for the models used to perform scenario exploration, and reported them in Table 1. These values and their calculation are discussed at lines 99-104 and 307-310 of the revised manuscript.

6. *Mechanistic interpretation. While the discussion speculates on mechanisms such as thermal performance curves and match–mismatch dynamics, these remain untested. Without independent evidence, such mechanisms should be framed more cautiously, or ideally tested through complementary modeling approaches.*

We agree with the reviewer, and have both tempered our discussion of such mechanisms, and better embedded them in the scientific literature at lines 193-203 of the revised manuscript.

*Minor Comments*

7. *Terminology. Ensure consistent use of “drivers,” “covariates,” and “indices,” as these terms are occasionally interchanged.*

We have adjusted our use of terminology throughout to refer to all candidate time series examined via convergent cross-mapping as covariates, and covariates with significant cross-mapping skill as drivers.

8. *Figures. Several figures are complex and could benefit from clearer labelling (e.g., distinguishing pre- and post-collapse periods more explicitly).*

We have recolored and replotted Figures 2 and 3 in an attempt to clarify their legends, and edited their captions for clarity. We are open to continuing to improve the clarity of our figures given the advice of the reviewers.

9. *Table 1. Consider highlighting significant CCM results more visually (e.g., bold or shading), as the asterisks are easy to miss.*

We agree with the reviewer and have bolded significant results in Table 1.

10. *Citations. Some references to previous studies (e.g., cod–capelin bottom-up control) could be expanded to situate results more firmly in the existing literature.*

We have expanded on our references to bottom-up control of cod by capelin availability at lines 171-178.

11. *Language. The manuscript is generally clear, but occasional phrasing (e.g., “likely statistical artifacts”) could be expressed with more academic precision*

We have attempted to clarify such phrases throughout the revised manuscript.
