# Supplementary material for: Identifying and assessing nonlinear drivers of capelin and Atlantic cod population dynamics using empirical dynamic modelling (EDM) scenario exploration
Source: PLoS One. 2025 Dec 19;20(12):e0339407. doi: 10.1371/journal.pone.0339407 (PMC12716747; doi:10.1371/journal.pone.0339407)
Supplement: Attachment — Submitted filename: Ch2_R2R2_F.docx [file pone.0339407.s004.docx]

Dear PLoS ONE Editorial Staff,

We appreciate your interest in our study, and your consideration for its publication in PLoS ONE. We appreciate the reviewer’s positive reception to our last iteration of the manuscript. We have resubmitted a new revised manuscript given the reviewer’s most recent set of comments. Here we address each comment and identify where it was addressed in the revised manuscript.

Sincerely,

R.W. Steele, J. Gao, M. Koen-Alonso, and P.M. Regular

1. *This version is a great improvement over the previous version I reviewed. Most of the comments have been addressed adequately. The authors have clarified that my main concern regarding over-parametrization turned out to be a misunderstanding. I would generally advise on being more explicit about this in the manuscript.*

We have attempted to clarify throughout the revised manuscript that all CCM and scenario exploration testing is univariate with respect to the number of drivers considered, with the exception of the multivariate scenario exploration example added in the previous revision.

2. *However, this clarification also raised another concern regarding multiple testing: a range of covariates were tested. This leads to increased probability of false positives, which I think can be addressed by some additional discussion (around line 245).*

We have added clearer mention of the issue of identifying false positives throughout the revised manuscript. As the reviewer suggests, we also add additional discussion regarding this issue at lines 248-254 of the revised manuscript.
